# Proteomic analysis of Drosophila CLOCK complexes identifies rhythmic interactions with SAGA and Tip60 complex component NIPPED-A

**DOI:** 10.1038/s41598-020-75009-5

**Published:** 2020-10-21

**Authors:** Guruswamy Mahesh, Gustavo B. S. Rivas, Courtney Caster, Evan B. Ost, Ravi Amunugama, Richard Jones, David L. Allen, Paul E. Hardin

**Affiliations:** 1 Department of Biology and Center for Biological Clocks Research, Texas A&M University, College Station, TX 77843 USA; 2 MS Bioworks, LLC, Ann Arbor, MI 48108 USA

**Keywords:** Biochemistry, Genetics, Molecular biology, Neuroscience

## Abstract

Circadian clocks keep time via ~ 24 h transcriptional feedback loops. In Drosophila, CLOCK-CYCLE (CLK-CYC) activators and PERIOD-TIMELESS (PER-TIM) repressors are feedback loop components whose transcriptional status varies over a circadian cycle. Although changes in the state of activators and repressors has been characterized, how their status is translated to transcriptional activity is not understood. We used mass spectrometry to identify proteins that interact with GFP-tagged CLK (GFP-CLK) in fly heads at different times of day. Many expected and novel interacting proteins were detected, of which several interacted rhythmically and were potential regulators of protein levels, activity or transcriptional output. Genes encoding these proteins were tested to determine if they altered circadian behavior via RNAi knockdown in clock cells. The NIPPED-A protein, a scaffold for the SAGA and Tip60 histone modifying complexes, interacts with GFP-CLK as transcription is activated, and reducing *Nipped-A* expression lengthens circadian period. RNAi analysis of other SAGA complex components shows that the SAGA histone deubiquitination (DUB) module lengthened period similarly to *Nipped-A* RNAi knockdown and weakened rhythmicity, whereas reducing *Tip60* HAT expression drastically weakened rhythmicity. These results suggest that CLK-CYC binds NIPPED-A early in the day to promote transcription through SAGA DUB and Tip60 HAT activity.

## Introduction

Daily rhythms in physiology, metabolism and behavior are found in a wide array of animals, plants and microbes. These rhythms are driven by endogenous circadian (~ 24 h) clocks, which keep time via cell-autonomous transcriptional feedback loops (TFL)^1^. In animals, core TFL components are well conserved, and drive rhythmic expression in 10–20% of genes in a given cell type that control overt rhythms in physiology, metabolism and behavior^2,3^. Environmental cycles of light, temperature and feeding entrain TFLs in different tissues to local time, thereby synchronizing rhythms so that they occur at the appropriate time of day to optimize energy use, reproduction and fitness.

In the fruit fly, *Drosophila melanogaster*, TFLs are activated when CLOCK-CYCLE (CLK-CYC) heterodimers bind E-box sequences to activate expression of *period* (*per*) and *timeless* (*tim*)^2,4^. PER-TIM complexes then accumulate, enter the nucleus, and bind CLK-CYC to repress their own gene’s transcription^2,4^. Once PER-TIM releases CLK-CYC from the E-boxes, CLOCKWORK ORANGE (CWO) binds to the E-boxes to maintain transcriptional repression until PER and TIM are degraded^5^, which permits CLK-CYC binding to displace CWO and initiate the next cycle of transcription^2,4^. CLK-CYC activation and PER-TIM repression are driven by rhythms in CLK, PER and TIM phosphorylation^2,4^. PER and TIM phosphorylation triggers their nuclear entry, where they engage CLK-CYC to promote CLK phosphorylation, release of CLK-CYC from the DNA, and chromatin modifications that repress transcription. Continued PER and TIM phosphorylation promotes their degradation, which results in CLK dephosphorylation, CLK-CYC binding to DNA, and chromatin modifications that activate transcription^2,4^. Although key regulators of CLK, PER and TIM phosphorylation, nuclear localization, and/or stability have been identified^2,4^, relatively little is known about how these processes promote transcriptional activation or repression.

In mammals, a similar feedback loop functions to keep circadian time: CLOCK-BMAL1 activates transcription of the Per1-3 and Cry1-2 genes, and PER-CRY complexes feedback to inhibit transcription until they are degraded, thus permitting the next cycle of CLOCK-BMAL1 activation^3^. As in flies, rhythmic transcription coincides with rhythms in clock protein phosphorylation and chromatin modifications^3^. PER-containing complexes contain multiple chromatin modifiers that repress transcription, including histone deacetylases^6^, helicases^7,8^, histone methyltransferases^9,10^, and nucleosome remodelers^11^. Factors that activate clock-dependent transcription have also been identified in mammals including MLL1 histone methyltransferase (HMT)^12^, *Jarid1a* histone demethylase (HDM)^13^, and histone acetyltransferases (HATs)^14,15^. In Drosophila, chromatin remodeling enzymes including the CBP/p300 HAT *nejire*^16^, the *Brahma* (*Brm*) nucleosome remodeler^17^, and the *dkDM2*, JMJD5, KDM3 and *lid* HDMs that regulate circadian behavior and/or CLK-CYC transcription^13,18,19^. Additionally, histone deubiquitination by the Spt-Ada-Gcn5 acetyltransferase (SAGA) complex also regulates *tim* and *Pdp1*ε transcription in Drosophila^20^. To understand how rhythmic transcription is regulated, factors that control the state of clock activation and repression complexes and translate the state of these complexes to set transcriptional activity must be identified and characterized.

To identify factors that regulate rhythmic transcription, we isolated CLK complexes from fly heads at different times of day and identified constituent proteins via tandem mass spectrometry (LC/MS/MS). CLK and CYC were present throughout the daily cycle, while PER and TIM levels varied over time with a peak coincident with transcriptional repression, thus validating the LC/MS/MS approach. Although many proteins were detected in CLK complexes, we focused our analysis on rhythmic CLK interactors that regulate protein phosphorylation, protein degradation or DNA templated transcription. Functional analysis of these CLK interactors via RNAi knockdown in all clock cells or PIGMENT DISPERSING FACTOR (PDF) expressing clock brain neurons shows that two novel interactors, protein phosphatase PRL-1 and chromatin modifying complex protein NIPPED-A, significantly lengthen the period of locomotor activity rhythms. Further analysis of NIPPED-A, a scaffold that links promoter bound transcription activators to the SAGA and Tip60 chromatin remodeling complexes^21,22^, revealed interactions with CLK-CYC when transcription transitions from repression to activation. RNAi knockdown of *Nipped-A* expression lengthened period, implying that the SAGA and/or Tip60 complexes contribute to CLK-CYC transcription. RNAi knockdown of SAGA complex HAT components modestly lengthened period, whereas RNAi knockdown of the Tip60 HAT drastically reduces rhythmicity. RNAi knockdown of SAGA histone deubiquitination (DUB) components lengthen period and/or disrupt rhythmicity. These results suggest that NIPPED-A engages the SAGA and Tip60 complexes to regulate CLK-CYC transcription via histone acetylation and deubiquitination.

## Results

### Identifying CLK associated proteins over a diurnal cycle

To isolate CLK complexes we employed transgenic *Clk*^*out*^ mutant flies that express GFP tagged CLK protein. The GFP-*Clk* transgene was previously shown to rescue strong rhythmicity in > 93% of *Clk*^out^ null flies with a period of 24.5 h^23^, thus GFP-CLK is fully functional. Flies entrained in a 12 h light:12 h dark (LD) cycle were collected when CLK-CYC transcription is activated at Zeitgeber Time 9 (ZT9) and ZT12 (Zeitgeber Time is time during an LD cycle where ZT0 is lights-on and ZT12 is lights-off), during the transition from active to repressed transcription at ZT16, during transcriptional repression at ZT20 and ZT0, and during the transition from repressed to active transcription at ZT6. Head extracts from these flies were used to immunoprecipitate (IP) CLK complexes using GFP nanobodies, while IP of flies expressing GFP in clock cells served as a negative control. Purified GFP and CLK-GFP complexes were size separated on SDS-PAGE and prepared for LC/MS/MS (see Experimental procedures). LC/MS/MS was carried out once for IP samples from each timepoint and analyzed for interacting proteins. CLK-associated proteins were defined as proteins having a minimum of 5 spectral counts (SpC) in the CLK-GFP sample that were not detected or had at least fourfold lower SpC in the GFP control. Based on these criteria 69–138 CLK-associated proteins were identified in the six CLK-GFP samples (Supplementary Data S1).

**Figure 1:**
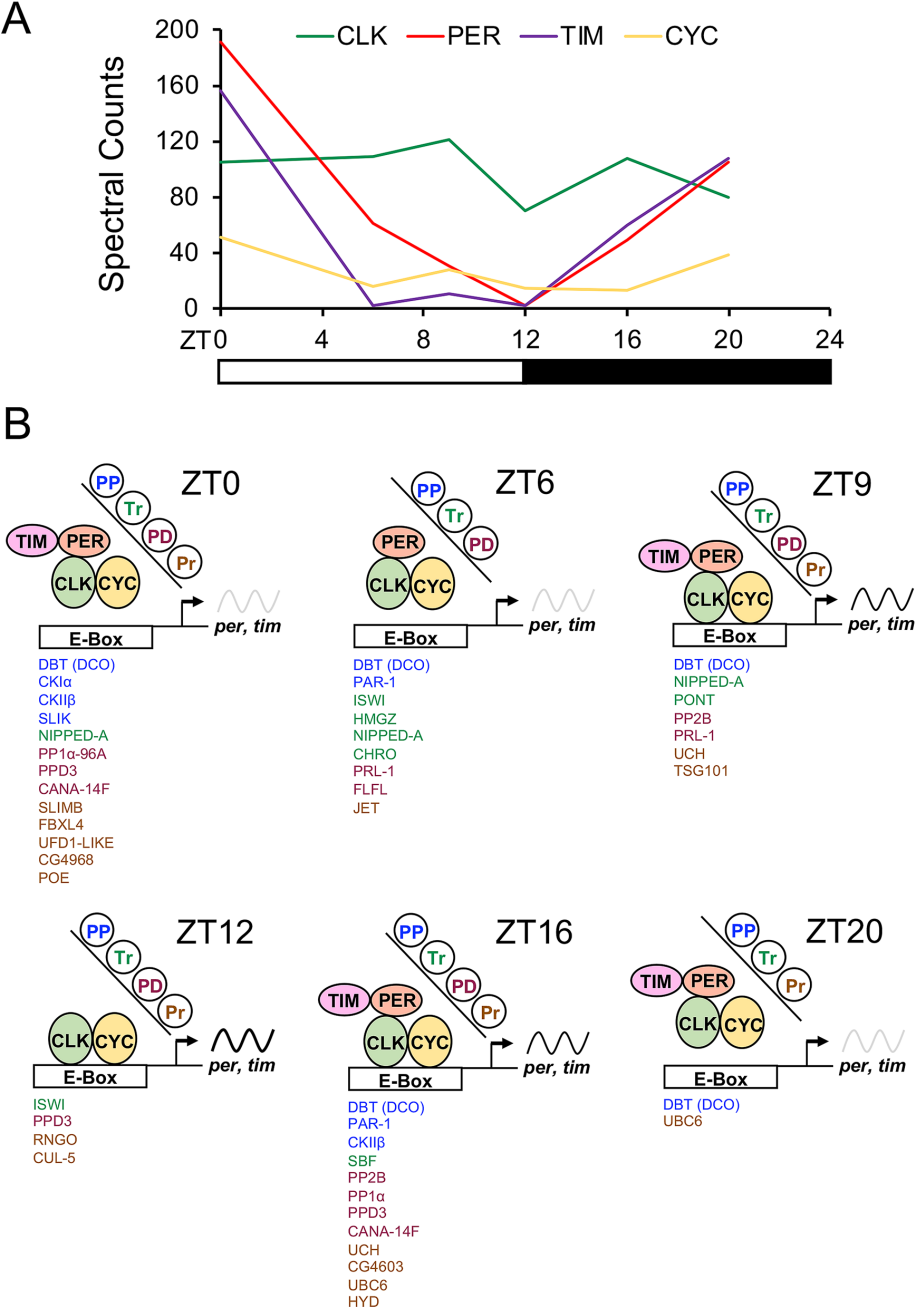
Proteins present in CLK complexes during a diurnal cycle. (**A**) Relative levels of the core clock proteins CLK (green), CYC (yellow), PER (red) and TIM (purple) in fly heads, as measured by the number of spectral counts obtained from MS/MS analysis of GFP-CLK complexes, is shown for flies collected at Zeitgeber Time (ZT) 0, ZT6, ZT9, ZT12, ZT16 and ZT20. White bar, times when lights are on; black bar, times when lights are off. (**B**) CLK complexes were isolated from the heads of flies collected at the indicated times. Proteins involved in protein phosphorylation (PP), transcription-DNA templated (Tr), protein dephosphorylation (PD), proteolysis (Pr) and circadian rhythms (e.g. CLK, CYC, PER, TIM) present in complexes at each time of day were identified as described (see “Materials and methods”). A list of CLK complex proteins in the PP (blue), Tr (green), PD (red) and Pr (brown) categories is listed for each collection time. The state of CLK-CYC transcription at each collection time is represented by the sinusoidal *per* and *tim* mRNA cycling line (low, light gray; moderate, dark gray; high, black) and whether CLK complexes are bound to E-boxes.

In each sample CLK was among the top three proteins based on SpC detected, with the number of SpC fluctuating between 70 and 121 (Fig. 1A). As expected, CYC was detected in each sample^24^, with relatively low but constant numbers of spectral counts throughout the daily cycle (Fig. 1A). PER spectral counts in CLK complexes changed dramatically over time from < 5 SpC at ZT12 to 191 SpC at ZT0. Likewise, TIM spectral counts also changed dramatically over time in CLK complexes from low levels (0–11 SpC) during the day (ZT6–ZT12) to high levels (156 SpC) at ZT0. Similar results were seen when PER, TIM and CYC spectral counts were plotted relative to CLK (Supplementary Figure S1). The number of spectral counts detected for core clock activators and repressors in CLK-GFP complexes over a diurnal cycle mirrors their abundance as determined previously on western blots, in clock cells and in clock complexes^25–31^, thus justifying the use of CLK-GFP IPs to identify CLK associated proteins.

In addition to the core feedback loop components, CLK-GFP complexes contained other proteins previously associated with clock function. DBT kinase, which contributes to PER stability and nuclear localization^32,33^, was detected at levels that co-varied with PER (Supplementary Data S1). The E3 ubiquitin ligase SLIMB promotes PER degradation in the nucleus^34–36^, and is only detected at ZT24, coincident with the initiation of PER degradation. Casein kinase II, which contributes to PER nuclear localization, PER and CLK stability and CLK repression^37–39^, is detected at ZT16 and ZT24. Finally, the E3 ubiquitin ligase JETLAG, which targets TIM and the circadian photoreceptor CRYPTOCHROME (CRY) for degradation upon light exposure^40–42^, is detected at ZT6. Notably, neither SHAGGY (SGG) kinase, which promotes PER and TIM nuclear localization^43,44^, nor CRY are detected in CLK-GFP complexes. It is possible that CRY is largely eliminated by ZT6, and thus undetectable in CLK-GFP complexes. SGG necessarily acts on PER-TIM complexes in the cytoplasm, and even though cytoplasmic PER-TIM complexes could interact with nuclear CLK-GFP in whole cell extracts, such interactions are either uncommon or SGG interactions with PER-TIM are weak or transient. Several highly abundant cytosolic and extracellular structural proteins (i.e. actin, tubulin, cuticle proteins) were also found in CLK-GFP complexes, but not GFP complexes. Given these proteins are not present in the nucleus, they apparently non-specifically interact with the CLK portion of CLK-GFP in whole cell extracts.

To identify proteins in CLK-GFP complexes that regulate transcription, we focused on proteins that show time-specific complex association from the Gene Ontology (GO) groupings protein phosphorylation, protein dephosphorylation, proteolysis, and transcription-DNA templated (hereafter DT-transcription). Throughout a diurnal cycle 5 protein phosphorylation components (e.g. kinases), 8 protein dephosphorylation components (e.g. phosphatases), 13 proteolysis components, and 6 DT-transcription components were detected (Fig. 1B). Of these CLK-GFP interactors, 3 protein phosphorylation subunits (PAR-1, DBT, CKII), 3 protein dephosphorylation factors (PRL-1, PP2B-14D, PP1alpha-96A), 2 DT-transcription (NIPPED-A, ISWI) and 2 proteolysis components (UCH, UBC6) were detected at more than one time point, but only DBT, PRL-1, PPD3, NIPPED-A and UBC6 interacted time-specifically as defined by detection at two or more consecutive timepoints (Supplementary Data S1; Fig. 1B). DBT is a component of PER-TIM repression complexes that is found in CLK-GFP complexes coincident with PER (Supplementary Data S1). PPD3 function in the clock was tested previously, but RNAi knockdown of *Ppd3* did not alter circadian behavior^45^. The remaining GFP-CLK interactors, *Prl-1*, *Nipped-A* and *Ubc6*, were characterized in more detail.

### NIPPED-A associates with CLK-CYC and influences circadian period

To characterize *Prl-1*, *Nipped-A* and *Ubc6* function within the circadian clock, we tested whether knocking down their expression in clock cells via RNAi altered behavioral rhythms. To do this, gene-specific UAS-RNAis were expressed in all clock cells using the *tim*-Gal4 driver or the ventrolateral neuron (LN_v_) subset of brain pacemaker neurons using the *pdf*-Gal4 driver (see Experimental procedures). In addition, RNAi production was enhanced by expressing the RNAi processing enzyme DICER by including a UAS-*dicer* transgene^46^. RNAi knockdown flies (i.e. UAS-*dicer* + Gal4 + UAS-RNAi) were only considered period-altered if they had a significantly longer or shorter period (p < 0.05) than the RNAi only (i.e. UAS-RNAi) or Gal4 driver (UAS-*dicer* + Gal4) controls having the longest or shortest periods, respectively. Since none of the RNAi knockdown lines tested produced periods shorter than their RNAi only or Gal4 driver controls, only periods significantly longer than the control with the longest period will be reported. Likewise, RNAi knockdown flies having a power that was significantly lower (p < 0.05) than the RNAi only or Gal4 driver controls having the lowest rhythm power were considered weakly rhythmic. Driving *Ubc6* RNAi with *tim*-Gal4 or *pdf*-Gal4 produced rhythms with periods of 24.43 h and 23.81 h, respectively, which were not significantly longer than their respective Gal4 driver controls (Table 1). Driving *Prl-1* RNAi with *pdf*-Gal4 lengthened period to 26.36 h, which was significantly (p < 10^–3^) longer than the *pdf*-Gal4 control. *Prl-1* RNAi driven by *tim*-Gal4 lengthened period to 24.97 h, but period in these flies was highly variable (s.e.m. = 1.84 h) and not significantly different (p = 0.58) than the *tim*-Gal4 control (Table 1). Both *tim*-Gal4 and *pdf*-Gal4 driven *Prl-1* RNAi flies had significantly reduced power (p < 10^–6^), which coincided with a lower % rhythmicity. *Nipped-A* RNAi driven by *tim*-Gal4 or *pdf*-Gal4 lengthened period to 26.06 h and 26.49 h, respectively, which are significantly longer (p < 10^–6^) than their respective Gal4 controls (Table 1). These data demonstrate that clock cell-specific RNAi knockdown of *Prl-1* and *Nipped-A*, but not *Ubc6*, either significantly lengthens circadian period or reduces rhythm power and % rhythmicity. Drosophila *Prl-1* is a member of the PRL family of tyrosine phosphatases, which function in tissue growth and tumor progression^47^, whereas NIPPED-A is an adapter subunit in the SAGA and Tip60 complexes that function primarily to co-activate transcription^21,22,48^.

**Table 1:** Activity rhythms of RNAi knockdown lines that target *Ubc6*, *Prl-1* and *Nipped-A.*

Genotype	Total	% Rhythmic	Period ± s.e.m	Strength ± s.e.m
*w*^1118^	16	100	23.64 ± 0.06	721.71 ± 74.91
*w*^1118^, UAS-*dcr*; *tim*-Gal4/ + ; + / +	15	100	24.50 ± 0.13	673.16 ± 64.50
*w*^1118^, UAS-*dcr*; + / + ; *pdf*-Gal4/ +	16	100	24.60 ± 0.08	680.41 ± 58.88
*w*^1118^, UAS-*dcr*; *Ubc6* RNAi^a^/*tim*-Gal4; + / +	28	100	24.43 ± 0.12	201.61 ± 25.27
*w*^1118^, UAS-*dcr*; *Ubc6* RNAi/ + ; *pdf*-Gal4/ +	17	100	23.81 ± 0.05	340.92 ± 45.96
*w*^1118^; *Ubc6* RNAi/ + ; + / +	19	100	23.22 ± 0.20	245.08 ± 39.25
*w*^1118^, UAS-*dcr*; *tim*-Gal4/ + ; *PRL-1* RNAi^b^/ +	9	44	24.97 ± 1.84	12.18 ± 4.19^4^
*w*^1118^, UAS-*dcr*; + / + ; *PRL-1* RNAi/*pdf*-Gal4	16	75	26.36 ± 0.58^1^	109.78 ± 47.53^5^
*w*^1118^; + / + ; *PRL-1* RNAi/ +	15	100	23.38 ± 0.06	866.26 ± 92.22
*w*^1118^, UAS-*dcr; Nipped-*A RNAi1^c^/*tim*-Gal4; + / +	14	93	26.06 ± 0.13^2^	295.93 ± 49.94
*w*^1118^, UAS-*dcr*; *Nipped-*A RNAi1/ + ; *pdf*-Gal4/ +	14	93	26.49 ± 0.23^3^	283.61 ± 48.25
*w*^1118^; *Nipped-A* RNAi1/ + ; + / +	16	94	24.00 ± 0.22	329.50 ± 60.17

Activity rhythm period in constant darkness is given in hours ± standard error of the mean (s.e.m.).

^a^*Ubc6* RNAi, BDSC# 42,631.

^b^*PRL-1* RNAi, BDSC# 38,358.

**Figure 2:**
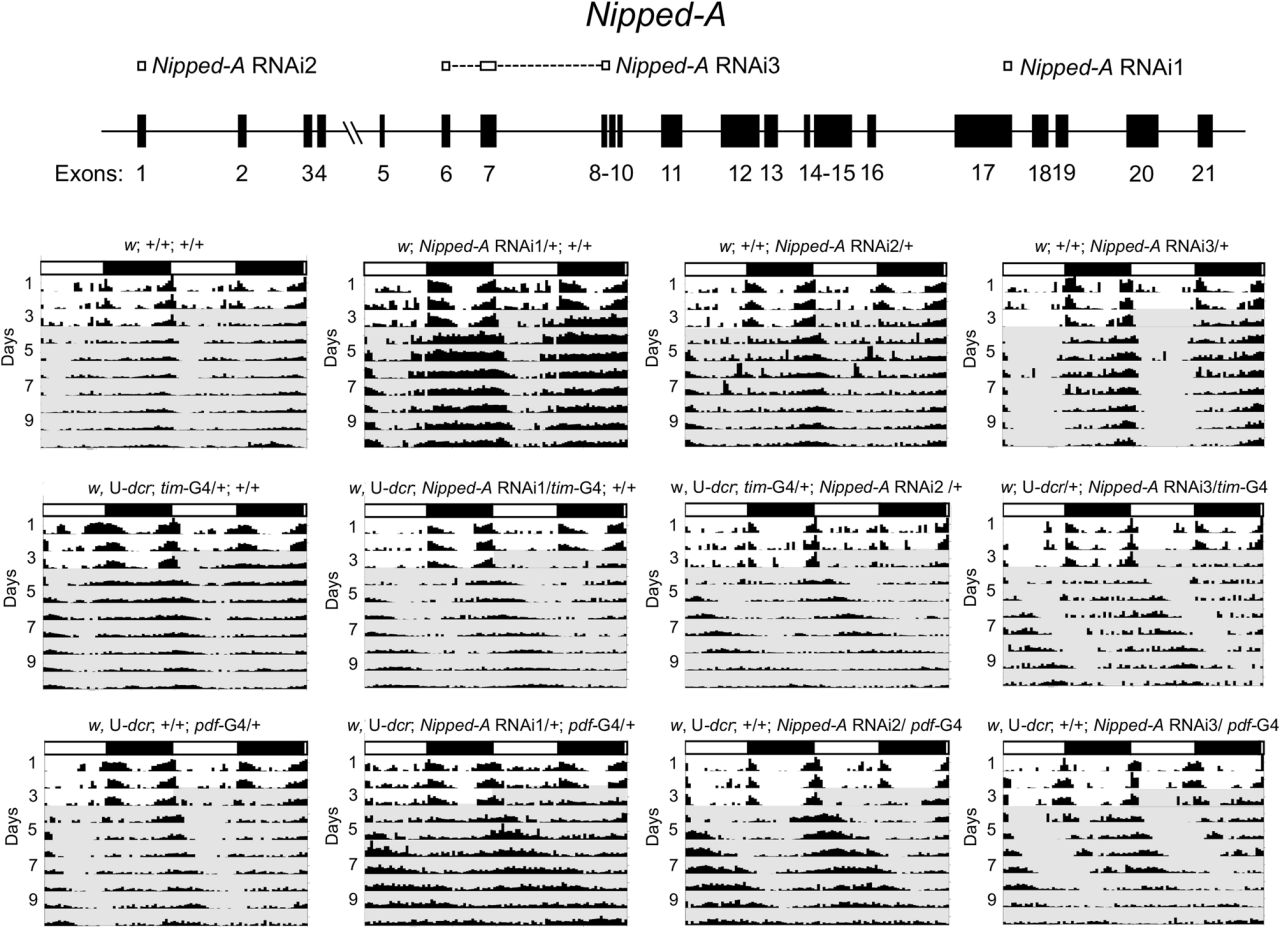
RNAi knockdown of *Nipped-A* lengthens circadian period. (**A**) Diagram showing the exon–intron structure of *Nipped-A* and the exons targeted by RNAis. *Nipped-A* exons (black boxes) are labeled 1–21. Exons targeted by *Nipped-A* RNAi1 (VDRC RNAi GD9847), *Nipped-A* RNAi2 (VDRC RNAi GD15595) and *Nipped-A* RNAi3 (NIG RNAi 2905R7) are shown as white boxes. (**B**) Representative actograms from the indicated genotypes. Flies were entrained, monitored and analyzed as described (see “Materials and methods”). White rectangles, light phase; black rectangles, dark phase; shaded background, activity during constant darkness; UAS-*dicer*, U-*dcr*; *tim*-Gal4, *tim*-G4; *pdf*-Gal4, *pdf*-G4.

^c^*Nipped-*A RNAi1, VDRC #GD9847. The sites targeted by *Nipped-*A RNAi1 is described in Fig. 2.

^1^Significantly longer than *w*^1118^, UAS-*dcr*; + / + ; *pdf*-Gal4/ + control flies (p < 10^–3^).

^2^Significantly longer than *w*^1118^, UAS-*dcr*; *tim*-Gal4/ + ; + / + control flies (p < 10^–6^).

^3^Significantly longer than *w*^1118^, UAS-*dcr*; + / + ; *pdf*-Gal4/ + control flies (p < 10^–8^).

^4^Significantly lower power than *w*^1118^, UAS-*dcr*; *tim*-Gal4/ + ; + / + control flies (p < 10^–7^).

^5^Significantly lower power than *w*^1118^, UAS-*dcr*; + / + ; *pdf*-Gal4/ + control flies (p < 10^–6^).

Given the direct link between *Nipped-A* and transcriptional activation, we characterized *Nipped-A* in more detail. To verify that reducing *Nipped-A* expression in clock cells lengthens period, RNAis targeting different parts of *Nipped-A* mRNA than exon 17, site of *Nipped-A* RNAi1 binding, were used to knock down expression in clock cells (Fig. 2). A UAS-*Nipped-A* RNAi line targeting exon 1 (*Nipped-A* RNAi2) lengthened period to 27.20 h with *tim*-Gal4 and 25.81 h with *pdf*-Gal4, which was significantly longer (p < 10^–11^) than their respective *tim*-Gal4 and *pdf*-Gal4 controls (Fig. 2; Table 2). Likewise, a *Nipped-A* RNAi line targeting exons 6–9 (*Nipped-A* RNAi3) lengthened period to 26.31 h with *tim*-Gal4 and 25.51 h with *pdf*-Gal4, which was significantly longer (p < 10^–3^) than *tim*-Gal4 and *pdf*-Gal4 controls, respectively (Fig. 2; Table 2). These results were comparable to the original GD9845 *Nipped-A* RNAi line that lengthened period to 26.06 h when driven by *tim*-Gal4 and 26.49 h when driven by *pdf*-Gal4 (Table 1; Fig. 2). The consistent long period phenotypes produced by driving different *Nipped-A* RNAis in clock cells is consistent with previous results^20^, thus confirming that *Nipped-A* contributes to circadian timekeeping.

**Table 2:** Activity rhythms of RNAi knockdown lines that target different portions of *Nipped-A* transcript.

Genotype	Total	% Rhythmic	Period ± SEM	Strength ± SEM
*w*^1118^	16	100	23.46 ± 0.05	630.71 ± 68.88
*w*^1118^, UAS-*dcr*; *tim*-Gal4/ + ; + / +	16	100	24.45 ± 0.09	331.74 ± 51.95
*w*^1118^, UAS-*dcr*; + / + ; *pdf*-Gal4/ +	14	100.00	24.06 ± 0.10	402.85 ± 71.09
*w*^1118^, UAS-*dcr*; *tim*-Gal4/ + ; *Nipped-*A RNAi2^a^/ +	12	91.66	27.20 ± 0.20^1^	300.95 ± 47.67
*w*^1118^, UAS-*dcr*; *Nipped-*A RNAi2/ *pdf*-Gal4	15	100.00	25.81 ± 0.19^2^	462.04 ± 42.47
*w*^1118^; *Nipped-*A RNAi2/ +	16	87.50	24.08 ± 0.12	366.06 ± 50.62
*w*^1118^, UAS-*dcr*; *Nipped-*A RNAi3^b^/ *tim*-Gal4	15	100.00	26.31 ± 0.12^3^	300.64 ± 43.49
*w*^1118^, UAS-*dcr*; + / + ; *Nipped-*A RNAi3/ *pdf*-Gal4	13	100.00	25.51 ± 0.39^4^	247.20 ± 37.26
*w*^1118^; *Nipped-*A RNAi3/ +	16	100.00	23.89 ± 0.03	1008.67 ± 125.11

Activity rhythm period in constant darkness is given in hours ± standard error of the mean (s.e.m.).

^a^*Nipped-*A RNAi2, VDRC #GD15595.

^b^*Nipped-*A RNAi3, NIG# 2905R-7. The sites targeted by *Nipped-*A RNAi2 and *Nipped-*A RNAi3 are described in Fig. 2.

^1^Significantly longer than *w*^1118^, UAS-*dcr*; *tim*-Gal4/ + ; + / + control flies (p < 10^–30^).

^2^Significantly longer than *w*^1118^, UAS-*dcr*; + / + ; *pdf*-Gal4/ + control flies (p < 10^–11^).

^3^Significantly longer than *w*^1118^, UAS-*dcr*; *tim*-Gal4/ + ; + / + control flies (p < 10^–33^).

^4^Significantly longer than *w*^1118^, UAS-*dcr*; + / + ; *pdf*-Gal4/ + control flies (p < 10^–3^).

**Figure 3:**
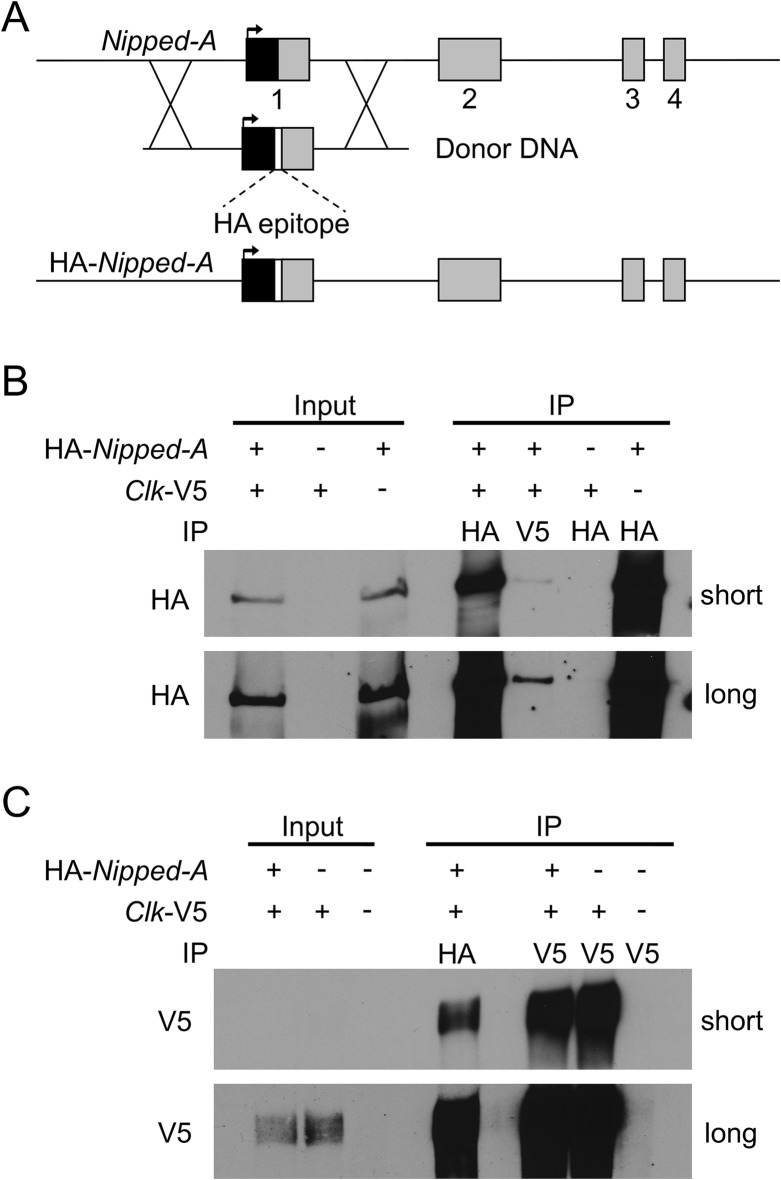
NIPPED-A interacts with CLK in S2 cells. (**A**) Diagram showing the first four exons of *Nipped-A*. A donor DNA sequence containing *Nipped-A* Exon 1 and flanking sequences was modified to contain an N-terminal HA epitope tag. A guide RNA for CRISPR/Cas9 situated near the start of translation was used to generate a dsDNA break used to replace the endogenous exon 1 sequences in S2 cells with the donor sequence (see “Materials and methods”) to generate the edited HA-*Nipped-A* gene. Arrow, transcription start; 5′UTR, black box; coding sequence, gray boxes; HA epitope, white box; X’s between donor and *Nipped-A*, regions of homologous recombination to replace *Nipped-A* sequence with donor sequence. (**B**) Proteins from HA-*Nipped-A* (+) or control non-tagged *Nipped-A* (−) S2 cells transfected with pMT-*Clk*-V5 ( +) or control pMT-V5 vector (−) plasmids were subjected to immunoprecipitation (IP) using V5 or HA antibodies. Westerns containing Input and IP samples were probed with HA antibody, and blots were exposed for 60 (short) or 120 (long) seconds then cropped to show reactive bands. (C) Proteins from HA-*Nipped-A* (+) or control non-tagged *Nipped-A* (−) S2 cells transfected with pMT-*Clk*-V5 (+) or control pMT-V5 vector (−) plasmids were subjected to IP using V5 or HA antibodies. Westerns containing Input and IP samples were probed with V5 antibody, and blots were exposed for 10 (short) or 60 (long) seconds then cropped to show reactive bands.

**Figure 4:**
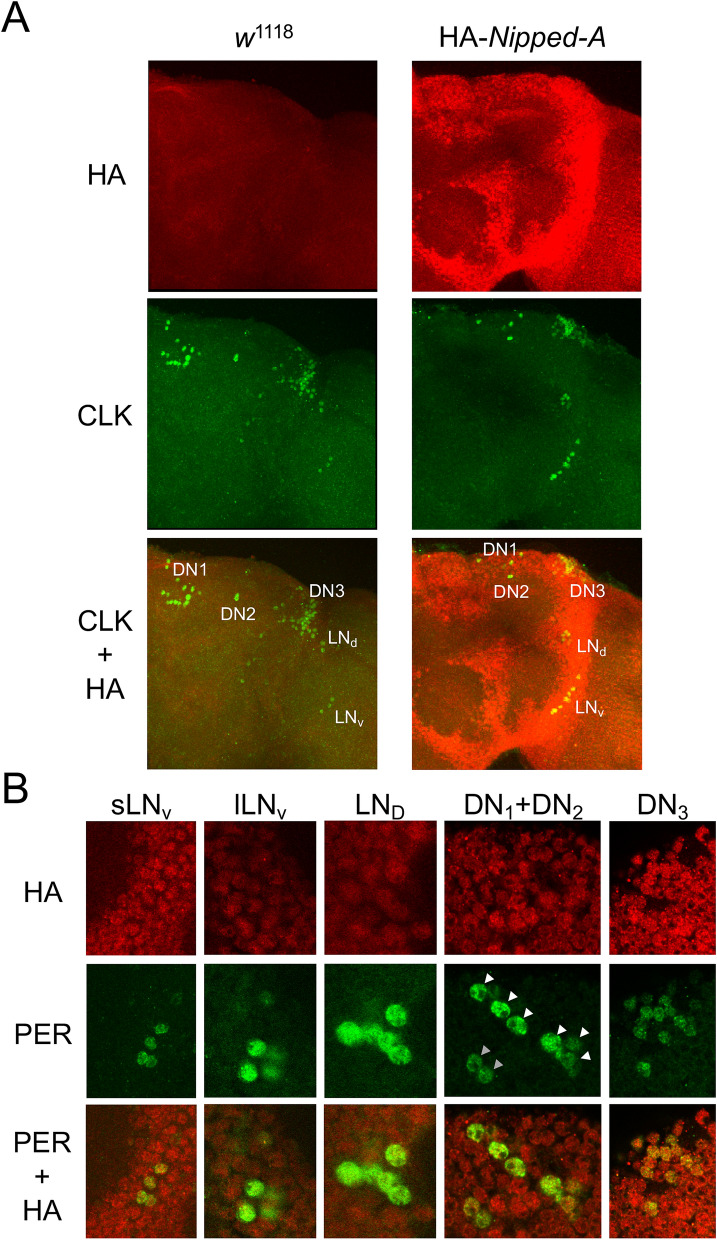
NIPPED-A is widely expressed in the brain including circadian pacemaker neurons. (**A**) Brains from *w*^1118^; + / + ; + / + (*w*^1118^) and *w*^1118^; HA-*Nipped-A*; + / + (HA-*Nipped-A*) flies collected at CT24 were immunostained with CLK and HA antibodies and imaged by confocal microscopy (see “Materials and methods”). CLK (green), HA (red) and CLK + HA (yellow) staining is shown for a right brain hemisphere, where lateral is right and dorsal is top. (**B**) Brains from HA-Nipped-A flies collected at CT24 were immunostained with HA and PER antibodies and imaged by confocal microscopy as in A. PER (green), HA (red) and PER + HA (yellow) signals are shown for the small ventrolateral neuron (sLN_v_), large ventrolateral neuron (lLN_v_), dorsolateral neuron (LN_d_), Dorsal Neuron 1 (DN_1_, white arrows), Dorsal Neuron 2 (DN_2_, gray arrows) and Dorsal Neuron 3 (DN_3_) groups of circadian pacemaker neurons. All images represent 8 or more brain hemispheres.

To independently confirm that NIPPED-A interacts with CLK-CYC, we tested for co-IP between NIPPED-A and CLK-CYC in Schneider 2 (S2) cells and in vivo. Because no antibodies to NIPPED-A are available, we epitope tagged endogenous *Nipped-A* in S2 cells at the N-terminus with 3xHA using CRISPR/Cas9 (Fig. 3a; see Experimental procedures). S2 cells express CYC but not CLK^49^, thus a plasmid that expresses V5 tagged CLK upon heavy metal induction (pMT-CLK-V5-His) was transfected into HA-NIPPED-A S2 cells to generate CLK-CYC heterodimers. V5 antibody was used to IP CLK-V5 complexes from these cells, which were probed with HA antibody to reveal the presence of HA-NIPPED-A (Fig. 3b; Supplementary Figure S2). When HA-NIPPED-A S2 cells transfected with pMT-CLK-V5 were IPed with HA antibody, CLK-V5 was detected in these complexes (Fig. 3c, Supplementary Figure S2). These experiments indicate that CLK interacts with NIPPED-A in S2 cells, however one caveat to these results is the lack of negative controls demonstrating that V5 antibody doesn’t non-specifically IP a band the same size as NIPPED-A (Fig. 3b, Supplementary Figure S2) and HA antibody doesn’t non-specifically IP a band the same size as CLK (Fig. 3c, Supplementary Figure S2). Despite this caveat, these results are consistent with previous results showing that NIPPED-A is associated with CLK in S2 cells^50^. To determine if NIPPED-A interacts with CLK in vivo, we used CRISPR to knock-in an N-terminal HA tag to the *Nipped-A* gene (see Experimental procedures). Immunostaining brains of HA-NIPPED-A and wild-type flies with HA and CLK antibodies revealed that HA-NIPPED-A is widely expressed in the brain, including regions containing clock neurons (Fig. 4a). To test whether HA-NIPPED-A is specifically detected by HA antibody, RNAi was used to knock down HA-*Nipped-A* expression in clock cells. HA-NIPPED-A expression was reduced in sLN_v_ and LN_d_ neurons marked by PER only in flies expressing *Nipped-A* RNAi, but not in controls lacking *Nipped-A* RNAi expression (Supplementary Figure S3). When brains from HA-*Nipped-A* flies were co-stained with PER and HA antibodies, HA and PER signals overlapped for all clock neurons (Fig. 4b), confirming that NIPPED-A is expressed in the brain pacemaker neurons. Despite the presence of HA-NIPPED-A in clock cells, no interactions between CLK and HA-NIPPED-A were detected in fly heads by IP using either HA-NIPPED-A or CLK (data not shown). Since CLK is only present in a small fraction of NIPPED-A expressing cells, and likely competes with many other transcription factors for NIPPED-A binding, CLK-NIPPED-A interactions may be too dilute to detect by co-IP in vivo. To confirm that NIPPED-A interacts with CLK-CYC in vivo, flies expressing FLAG-tagged CYC were used to IP CLK-CYC complexes followed by more sensitive detection via LC/MS/MS^51^. FLAG-CYC was IPed from the heads of w; FLAG-*cyc*; *cyc*^01^ flies collected at ZT24, a time when NIPPED-A is present in CLK complexes (Supplementary Data S1; Fig. 1). Like CLK-GFP complexes at ZT24, FLAG-CYC complexes contained the expected core clock proteins CLK, PER and TIM (Supplementary Data S2). Importantly, FLAG-CYC complexes also detected NIPPED-A (Supplementary Data S2), thus confirming the presence of NIPPED-A in CLK-CYC complexes in vivo.

### NIPPED-A functions in the SAGA complex to regulate transcriptional processes

Given that NIPPED-A interacts with CLK-CYC as transcription transitions from repression (ZT0) to activation (ZT6, 9) and decreasing CLK-CYC lengthens circadian period^52–54^, we hypothesize that knocking down *Nipped-A* lengthens period by decreasing CLK-CYC transcription. The SAGA and Tip60 co-activator complexes both contain NIPPED-A and remodel chromatin via histone acetylation to activate transcription^21,22,48^. To determine whether the impact of *Nipped-A* on circadian rhythms stems from altered histone acetylation by the SAGA and/or Tip60 complexes, we tested whether RNAi knockdown of SAGA and Tip60 components altered activity rhythms. The SAGA complex acetylates histone H3 primarily at K9 and K14 residues via a HAT module containing Transcriptional Adaptor 2B (ADA2B), SAGA Associated Factor 29 kDa (SGF29) and the HAT GCN5, whereas the Tip60 complex acetylates histone H4 via the HAT TIP60^21,48^. SAGA and Tip60 complex components are widely expressed in Drosophila^55^, thus testing RNAi efficacy via RT-qPCR would not provide useful information since clock cells only comprise a small proportion of all cells in the fly head^56^. As an alternative, if RNAi knockdown of different SAGA HAT module components in clock cells all produced a consistent circadian phenotype, we considered that as validation of their efficacy. RNAi knockdown of *Ada2b*, *Sgf29* and *Gcn5* with *tim*-Gal4 or *pdf*-Gal4 each modestly lengthened period from 0.3 to 1.1 h (Table 3), suggesting that H3 acetylation makes some contribution to period lengthening. In contrast, RNAi knockdown of *Tip60* using *tim*-Gal4 or *pdf*-Gal4 did not alter circadian period, but significantly (p < 0.05) decreased rhythm power from control levels (214–706) to ~ 20–30 and reduced rhythmicity by ~ 50% (Table 3). This reduction in rhythm power and rhythmicity likely stems from Tip60 complex function in the clock since similar phenotypes result from *Tip60* RNAi knockdown in all clock cells or only LN_v_s. Taken together, these results suggest that *Nipped-A* alters circadian behavior via SAGA-dependent H3 acetylation and Tip60-dependent H4 acetylation.

**Table 3:** Activity rhythms of RNAi knockdown lines that target components of SAGA, Tip60 and Xmas-2 complexes.

Genotype	Total	% Rhythmic	Period ± SEM	Strength ± SEM
*w*^1118^	16	81	23.50 ± 0.09	459.00 ± 93.23
*w*^1118^, UAS-*dcr*; *tim*-Gal4/ + ; + / +	16	100	24.63 ± 0.15	679.73 ± 83.96
*w*^1118^; UAS-*dcr*/ + ; *tim*-Gal4/ +	16	100	24.33 ± 0.08	690.68 ± 67.32
*w*^1118^, UAS-*dcr*; + / + ; + /*pdf*-Gal4	16	100	24.60 ± 0.06	706.62 ± 104.19
*w*^1118^, UAS-*dcr*; *tim*-Gal4/ + ; *Ada2b* RNAi^a^/ +	16	93.75	25.44 ± 0.20^1^	246.41 ± 37.95
*w*^1118^, UAS-*dcr*; + / + ; *Ada2b* RNAi/*pdf*-Gal4	16	93.75	25.46 ± 0.24^2^	222.36 ± 47.13
*w*^1118^; + / + ; *Ada2b* RNAi/ +	16	100.00	24.12 ± 0.10	416.14 ± 48.76
*w*^1118^, UAS-*dcr*; *Sgf29* RNAi^b^/*tim*-Gal4; + / +	16	100.00	24.96 ± 0.21	325.04 ± 54.82
*w*^1118^, UAS-*dcr*; *Sgf29* RNAi/ + ; + /*pdf*-Gal4	15	93.33	25.12 ± 0.35	180.46 ± 50.50
*w*^1118^; *Sgf29* RNAi/ + ; + / +	16	100.00	23.78 ± 0.10	391.31 ± 37.40
*w*^1118^, UAS-*dcr*; *Gcn5* RNAi^c^/*tim*-Gal4; + / +	16	93.75	25.34 ± 0.33	155.12 ± 43.72
*w*^1118^, UAS-*dcr*; *Gcn5* RNAi/ + ; + /*pdf*-Gal4	10	90.00	24.77 ± 0.66	123.96 ± 48.11
*w*^1118^; RNAi *Gcn5* RNAi/ + ; + / +	15	100.00	23.60 ± 0.12	242.17 ± 29.91
*w*^1118^, UAS-*dcr*; *Tip60* RNAi^d^/*tim*-Gal4; + / +	30	40.00	25.35 ± 0.94	19.95 ± 5.14^7^
*w*^1118^, UAS-*dcr*; *Tip60* RNAi/ + ; + /*pdf*-gal4	32	53.12	24.30 ± 0.56	27.66 ± 6.14^8^
*w*^1118^; + / + ; *Tip60* RNAi/ +	31	96.77	23.92 ± 0.09	214.56 ± 23.35
*w*^1118^, UAS-*dcr*; *not* RNAi^e^/*tim*-Gal4; + / +	14	80.00	25.92 ± 1.13	48.27 ± 20.88
*w*^1118^, UAS-*dcr*; *not* RNAi/ + ; + /*pdf*-Gal4	14	78.57	26.42 ± 0.80^3^	42.38 ± 8.54
*w*^1118^; *not* RNAi/ + ; + / +	16	100.00	24.01 ± 0.08	198.57 ± 33.57
*w*^1118^, UAS-*dcr*; *e(y)2* RNAi^f^/*tim*-Gal4; + / +	16	87.50	26.12 ± 0.58^4^	52.88 ± 10.21^9^
*w*^1118^; UAS-*dcr*; *e(y)2* RNAi/ + ; + /*pdf*-Gal4	16	93.75	25.84 ± 0.42^5^	119.98 ± 27.07^10^
*w*^1118^; *e(y)2* RNAi/ + ; + / +	16	93.75	23.74 ± 0.14	397.22 ± 64.22
*w*^1118^, UAS-*dcr*; *xmas* RNAi^g^/*tim*-Gal4; + / +	14	92.85	24.84 ± 0.78	50.38 ± 6.66
*w*^1118^, UAS-*dcr*; + / + ; *xmas* RNAi/*pdf*-Gal4	31	100.00	23.90 ± 0.12	168.75 ± 22.44
*w*^1118^, *xmas* RNAi/ + ; + / +	16	93.75	23.47 ± 0.28	181.43 ± 40.95
*w*^1118^; UAS-*dcr*/ + ; *Sem1* RNAi^h^/*tim*-Gal4	2*	50	30.80 ± 0.00	15.12 ± 5.17
*w*^1118^, UAS-*dcr*; + / + ; *Sem1* RNAi/*pdf*-Gal4	16	81.25	25.65 ± 0.19^6^	156.36 ± 27.69
*w*^1118^; + / + ; *Sem1* RNAi/ +	16	93.75	24.24 ± 0.42	358.74 ± 52.93

Activity rhythm period in constant darkness is given in hours ± standard error of the mean (s.e.m.).

*14 of 16 flies died during the behavioral experiment.

^a^*Ada2b* RNAi1, NIG# 9638R-3.

^b^*Sgf29* RNAi, NIG# 10509R-1.

^c^*Gcn5* RNAi, BDSC# 9332.

^d^*Gcn5* RNAi, NIG 6121R1.

^e^*not* RNAi, NIG# 4166-R1.

^f^*e(y)2* RNAi; VDRC# KK102036.

^g^*xmas* RNAi, BDSC# 44427.

^h^*Sem1* RNAi, BDSC# 28057.

^1^Significantly longer than *w*^1118^, UAS-*dcr*; *tim*-Gal4/ + ; + / + control flies (p < 10^–2^).

^2^Significantly longer than *w*^1118^, UAS-*dcr*; + / + ; *pdf*-Gal4/ + control flies (p < 10^–2^).

^3^Significantly longer than *w*^1118^, UAS-*dcr*; + / + ; *pdf*-Gal4/ + control flies (p < 10^–2^).

^4^Significantly longer than *w*^1118^, UAS-*dcr*; *tim*-Gal4/ + ; + / + control flies (p < 0.05).

^5^Significantly longer than *w*^1118^, UAS-*dcr*; + / + ; *pdf*-Gal4/ + control flies (p < 10^–2^).

^6^Significantly longer than *w*^1118^, UAS-*dcr*; + / + ; *pdf*-Gal4/ + control flies (p < 0.05).

^7^Significantly lower power than *w*^1118^, UAS-*dcr*; *tim*-Gal4/ + ; + / + control flies (p < 0.05).

^8^Significantly lower power than *w*^1118^, UAS-*dcr*; + / + ; *pdf*-Gal4/ + control flies (p < 10^–2^).

^9^Significantly lower power than *w*^1118^, UAS-*dcr*; *tim*-Gal4/ + ; + / + control flies (p < 10^–2^).

^10^Significantly lower power than *w*^1118^, UAS-*dcr*; + / + ; *pdf*-Gal4/ + control flies (p < 0.05).

NIPPED-A also functions within the SAGA complex to activate transcription by binding the DUB module^21,48,57^. The DUB module contains SAGA ASSOCIATED FACTOR 11 kDa (SGF11), ATAXIN 7 (ATX7), ENHANCER OF YELLOW 2 (EY2) and the deubiquitinase NON-STOP (NOT)^21,57^. To determine if the DUB module contributes to clock function, we tested whether RNAi knockdown of DUB module components *not* and *e(y)2* in clock cells altered behavioral rhythms. RNAi knockdown of *not* with *tim*-Gal4 or *pdf*-Gal4 drivers lengthened period to 25.92 h and 26.42 h, respectively (Table 3), but was only significant (p < 10^–2^) for the *pdf*-Gal4 driver. Periods were highly variable for both *tim*-Gal4 driven (s.e.m. = 1.13 h) and *pdf*-Gal4 driven (s.e.m. = 0.80 h) *not* RNAi, and both the % rhythmicity and rhythm power were lower in *not* RNAi knockdown flies (Table 3). RNAi knockdown of *e(y)2* showed strong period lengthening to 26.12 h with *tim*-Gal4 and 25.84 h with *pdf*-Gal4, which was accompanied by somewhat reduced rhythm strength (Table 3). The period lengthening due to *not* or *e(y)2* RNAi knockdowns was similar in magnitude to that of *Nipped-A* RNAi knockdowns (Tables 1, 2), suggesting that *Nipped-A* function in the clock is largely mediated by the SAGA DUB module. These results are consistent with previous analysis of clock cell-specific *Nipped-A* and *not* RNAi knockdown, which showed long period rhythms, increased H2B ubiquitination and decreased transcription at CLK-CYC target genes *tim* and *Pdp1*ε^20^.

In addition to its function in the DUB module, *e(y)2* is also required for mRNA export from the nucleus as part of the Trex-2 complex^21,58^. To determine if the Trex-2 complex also contributes to clock function we tested whether knocking down expression of Trex-2 components *xmas*, which acts as a scaffold for Trex-2 complex^59^, and *Sem1*, which acts to stabilize Trex-2 complex^60^, in clock cells altered circadian rhythms. RNAi knockdown of *xmas* with *tim*-Gal4 and *pdf*-Gal4 didn’t significantly alter period, though *tim*-Gal4 driven *xmas* RNAi reduced rhythm strength (Table 3). Driving *Sem1* RNAi with *tim*-Gal4 caused a high level of lethality (14 of 16 flies tested), where one of the two remaining flies had a weak long period rhythm (Table 3). RNAi knockdown of *Sem1* with *pdf*-Gal4 significantly lengthened period to 25.65 h, along with modest decrease in rhythm power. In addition to its role in mRNA export within the Trex-2 complex, *Sem1* is also a component of the 26S proteasome involved in proteasome biogenesis^61^. Given that clock proteins including PER and TIM are degraded in the 26S proteasome and knocking down *xmas* expression in clock cells did not significantly alter behavioral rhythms (Table 3), the long period phenotype of *Sem1* RNAi knockdown flies may be due to decreased proteasome levels and/or activity.

## Discussion

Rhythmic transcription in Drosophila is activated by CLK-CYC complexes and repressed by PER-TIM complexes, but how daily changes in the activity of these complexes is controlled and translated to transcriptional activity is not well understood. We assessed GFP-CLK complexes at different times during a diurnal cycle to identify proteins that mediate transcriptional activation or repression via LC/MS/MS. Since CLK (and GFP-CLK) is predominantly nuclear at all times during the circadian cycle^23,62,63^, we expected interacting proteins would be nuclear proteins. However, in each IP GFP-CLK interactors included cytosolic or membrane-bound proteins (e.g. actin, tubulin, tropomyosin, mitochondrial proteins, cuticle proteins, translation factors and laminins). Our IP extracts were made using whole cells rather than nuclei, thus cytosolic factors could interact with GFP-CLK once nuclei were broken. Interactions between GFP-CLK and cytosolic or membrane-bound proteins are due to non-specific binding to CLK or proteins bound to CLK as these proteins were not detected in GFP controls (Supplementary Data S1). We consider GFP-CLK-interacting proteins that function in the nucleus as potential “true-interactors”, which may be a useful resource for future studies of clock function in Drosophila.

The core clock proteins CYC, PER and TIM are present in GFP-CLK complexes at the expected times of day (Fig. 1). Many other proteins are enriched in GFP-CLK complexes (Supplementary Data S1), and a small subset of these proteins associate with GFP-CLK at specific phases of the diurnal cycle. Of these proteins, DBT plays a prominent role in PER phosphorylation and degradation^33,64,65^ and PPD3 has no detectable impact on clock function^45^. RNAi knock down of *Prl-1* lengthened circadian period and decreased rhythm strength, while knocking down *Nipped-A* lengthened circadian period (Table 1), consistent with previous work^20^. We characterized *Nipped-A* further because of its known role in transcriptional regulation.

NIPPED-A functions as a scaffold that targets SAGA and Tip60 co-activator complexes to specific transcription factors^21,22^. The SAGA complex co-activates transcription via GCN5/PCAF-dependent H3K9/H3K14 acetylation or NOT-dependent H2B de-ubiquitylation, while the Tip60 complex co-activates transcription via TIP60-dependent H4/H2A/H2Az acetylation^21^. RNAi knockdown of SAGA HAT module components only partially accounts for the loss in SAGA function in clock cells when *Nipped-A* levels are reduced (Tables 1, 2), consistent with a lack of significant changes in H3K9 acetylation at the *per* and *Pdp1*ε loci in flies with reduced *Nipped-A* expression in clock cells^20^. Since H3K9 acetylation robustly cycles in concert with *per* and *tim* transcription in Drosophila^66^, either this histone modification has little impact on CLK-CYC transcription or a different HAT acetylates H3K9.

In contrast, knocking down SAGA DUB module components *e(y)2* and *not* lengthened circadian period to a similar extent as *Nipped-A* RNAi knockdown (Tables 1, 2, 3), consistent with RNA knockdown of *Sgf11*^20^, another component of the DUB module^21^. Decreased *not*, *e(y)2* and *Sgf11* expression in clock cells also significantly decreased rhythm power (Table 3)^20^, which is surprising given that reducing *Nipped-A* expression in clock cells didn’t significantly alter rhythm power (Tables 1, 2). Moreover, previous molecular analysis shows that reducing *Nipped-A* expression in clock cells increases H2B ubiquitination at two CLK-CYC target genes, *per* and *Pdp1*ε^20^. These genetic and molecular results suggest that the SAGA complex primarily contributes to circadian clock function via the DUB module.

Since NIPPED-A is also a core component of the Tip60 coactivator complex^21,22^, we knocked down *Tip60* HAT expression in clock cells and found that rhythm power was drastically reduced (Table 3). This reduction in power was unexpected because *Nipped-A* RNAi knockdown in clock cells didn’t significantly alter power (Tables 1, 2). Moreover, a primary target of TIP60 histone acetylation, histone H4, is not rhythmically acetylated at the *per* and *tim* loci in Drosophila^66^, but whether acetylation of TIP60 targets H2A and H2Az is rhythmic at these clock genes is not known. In mice, TIP60 acetylates BMAL1 (ortholog of Drosophila CYC) at K538 to promote CLOCK-BMAL1 transcription elongation^67^, but since CYC lacks the C-terminal region that would contain K538 such regulation can’t occur in Drosophila. Our results show that *Tip60* enhances the power of behavioral rhythms, presumably by maintaining the amplitude of molecular rhythms.

We identified *Nipped-A* based on its interaction with GFP-CLK during the transition from repressed to active transcription. The CLK-NIPPED-A interaction was confirmed in S2 cells via IP of HA-NIPPED-A and V5-CLK, consistent with previous mass spectrometry results in S2 cells^50^, and in vivo by LC/MS/MS of FLAG-CYC complexes (Fig. 3; Supplementary Data S2). Previous work suggested that NIPPED-A does not interact with CLK-CYC because it does not rhythmically interact with the *tim* and *Pdp1*ε loci coincident with CLK-CYC via ChIP^20^. However, the antibody used to detect NIPPED-A was raised against a poorly conserved (52% identical) peptide spanning residues 3809–3859 from human NIPPED-A ortholog TRRAP and was not validated for detecting Drosophila NIPPED-A. RNAi knockdown of *Nipped-A* in clock cells significantly reduced only *tim* and *Pdp1*ε mRNA levels, though *per* and *vri* mRNA levels were also reduced to some extent^20^. The variable effect of *Nipped-A* knockdown on *tim* and *Pdp1*ε versus *per* and *vri* transcripts could arise from differential use of NIPPED-A in the SAGA and Tip60 complexes. Since clock gene transcript abundance was measured in heads^20^, and the vast majority of clock gene expression in heads is from photoreceptors^56^, such differential use of NIPPED-A in the Tip60 and SAGA complexes could result from tissue-specific interactions with CLK-CYC complexes. Our data strongly support an interaction between NIPPED-A and the CLK-CYC complex, consistent with recent work showing that mammalian TRRAP is present in CLOCK-BMAL1 complexes in mice^7^. Thus, our results suggest that interactions between NIPPED-A and CLK-CYC promote histone modifications via the SAGA and Tip60 complexes, thereby translating CLK-CYC binding into target gene transcription.

In addition to the SAGA and Tip60 complexes, the CBP/p300 HAT *nej* and the HDMs *dkDM2*, JMJD5, KDM3 and *lid* regulate circadian clock function in Drosophila^13,16,18,19^. Like NIPPED-A, NEJ interacts directly with CLK-CYC and promotes CLK-CYC transcription^16^. Null mutants for the HDM genes *lid* and *KDM3* abolish behavioral rhythms and, in the case of *lid*, drastically reduce CLK-CYC transcription^13,18^. Likewise, the period shortening displayed by *dkDM2* and *JMJD5* mutants suggest that these HDMs act to repress CLK-CYC transcription^18,19^. Whether these HDM proteins function in CLK-CYC activator or PER-TIM repressor complexes is not known. However, the *Brahma* (*Brm*) nucleosome remodeling complex interacts with CLK-CYC to repress transcription through catalytic and non-catalytic activities^17^. Not surprisingly, many histone modifying/chromatin remodeling proteins appear to translate the state of circadian activation and repression complexes to control transcriptional activity.

## Materials and methods

### Fly strains

The *w*^1118^ and *w*^1118^;*CyO*/*Sco*;TM2/TM6B strains were used as a wild-type control for activity rhythms and as balancers to generate fly strains for analysis, respectively. The following mutants, Gal4 strains, epitope-tagged clock protein strains, GFP marker strain and RNAi efficacy enhancer strain were used in this study: *Clk*^out^^68^, *cyc*^*01*^^54^, *tim*-Gal4^62^ and *tim*-Gal4^16^^69^, *pdf*-Gal4^70^, CLK-GFP^23^, FLAG-*cyc*^51^, UAS-GFP::lacZ.nls30.1 (BDSC 6452) and UAS-*dicer*^46^. The following RNAi strains were obtained from the Vienna Drosophila Resource Center (VDRC), the National Institute of Genetics of Japan (NIG-FLY) or the Bloomington Drosophila Stock Center (BDSC): VDRC GD15595 (*Nipped-A*), VDRC GD9847 (*Nipped-A*), NIG 2905-R7 (*Nipped-A*), BDSC 42631 (*Ubc6*), BDSC 38358 (*PRL-1*), NIG-FLY 9638R-3 (*Ada2b*), NIG-FLY 10509R-1 (*Sgf29*), BDSC 9332 (*Gcn5*), NIG-FLY 4166R-1 (*not*), VDRC KK102036 (*e(y)2*), BDSC 44427 (*xmas*) and BDSC 28057 (*Sem1*).

### Purification of GFP-CLK complex

Fly head extracts were prepared from CLK-GFP and *tim*-Gal4 driven UAS-GFP.nls flies. Approximately 5 ml of fly heads were homogenized as described previously^71^. The extraction supernatant was incubated overnight at 4 °C with 50 μl of GFP- Nanobeads (ChromoTek). Beads were then washed with cold EB3 buffer three times for 30 min each time, and GFP bound beads were sent for the LC/MS/MS analysis.

### Mass spectrometry

Each sample was solubilized in LDS buffer, heated at 85 °C for 5 min and the full amount separated ~ 1.5 cm on a 10% Bis–Tris Novex mini-gel (Invitrogen) using the MES buffer system. The gel was stained with coomassie and each lane was excised into ten equally sized segments. Gel segments were reduced using dithiothreitol, alkylated with iodoacetamide and then subjected to digestion with trypsin (Promega). Digests were analyzed by nano LC/MS/MS with a NanoAcquity HPLC system (Waters, MA) interfaced to an LTQ Orbitrap Velos tandem mass spectrometer (ThermoFisher) as described^68^. Peptides were loaded on a trapping column and eluted over a 75 µm analytical column at 350 nL/min; both columns were packed with Jupiter Proteo resin (Phenomenex). A 30 min gradient was employed for each segment. The mass spectrometer was operated in data-dependent mode, with MS performed in the Orbitrap at 60,000 FWHM resolution and MS/MS performed in the LTQ at unit resolution. The fifteen most abundant ions were selected for MS/MS from each MS scan. Dynamic exclusion and repeat settings ensured each ion was selected only once and excluded for 30 s thereafter. Product ion data were searched against the combined forward and reverse Uniprot *D. melanogaster* protein database using a locally stored copy of the Mascot search engine v2.3 (Matrix Science, London, U.K.) via Mascot Daemon v2.3. Peak lists were generated using the Extract_MSn executable (ThermoFisher). The database was appended with common background proteins. Search parameters were precursor mass tolerance 10 ppm, product ion mass tolerance 0.5 Da, 2 missed cleavages allowed, fully tryptic peptides only, fixed modification of carbamidomethyl cysteine, variable modifications of oxidized methionine, protein N-terminal acetylation and pyro-glutamic acid on N-terminal glutamine. Mascot search result flat files (DAT) were parsed to the Scaffold software v3.1 (Proteome Software) with following cutoff values: 90% protein and 50% peptide level probability (probabilities were assigned by the Protein Prophet algorithm).

### Immuno-precipitation and Western blot analysis

S2 cell extracts were prepared using EB3 buffer as described^68^. The extract supernatant was collected, and immunoprecipitated using V5-beads (Sigma) or HA- beads (Sigma), the samples were resolved in 5% gel, transferred, and probed with rabbit anti-V5 (Sigma, 1:5000); anti-HA (Sigma 1:5000). Horseradish peroxidase-conjugated secondary antibodies (Sigma) were diluted 1:1000. Immunoblots were visualized using ECL plus reagent (GE Life Sciences).

### Immunostaining

Brains from *w*^1118^; + */* + ; 3xHA-*Nipped-A*, *w*^1118^, UAS-*dicer*; 3 × HA-*Nipped-A*/ + ; *Nipped-A* RNAi3/ + and *w*^1118^, UAS-*dicer*; 3 × HA-*Nipped-A*/*tim*-Gal4; *Nipped-A* RNAi3/ + flies were collected at ZT0, dissected, fixed, immunostained and imaged by confocal microscopy as described^62^. The primary antibodies used were anti-CLK GP50^62^ at a 1:3000 dilution, preabsorbed rabbit anti-PER antibody (a gift from Michael Rosbash) at a 1:20,000 dilution and mouse anti-HA antibody (Sigma #H9658) at a 1:1000 dilution. Secondary antibodies used were donkey anti-rabbit Alexa 488 (Jackson ImmunoResearch #711-545-152), donkey anti-mouse Alexa 488 (Jackson ImmunoResearch #715-545-150), donkey anti-Guinea pig Cy3 (Jackson ImmunoResearch #706-165-148) and donkey anti-mouse Cy3 (Jackson ImmunoResearch #715-165-150), all at a 1:200 dilution. Confocal stacks were imaged using an Olympus FV1000 confocal microscope equipped with 20 × 0.85 NA and 100 × 1.40 NA oil-immersion objectives as described^23^.

### Generation of HA-tagged *Nipped-A* coding sequence in S2 cells

S2 cells were maintained in Schneider’s Drosophila medium (Invitrogen) containing 10% fetal bovine serum with (100 U/ml) Penicillin and Streptomycin (100 μg/ml) (Invitrogen). An epitope tag consisting of three tandem hemagglutinin repeats (3xHA) was inserted at the N-terminus of *Nipped-A* in Schneider 2 (S2) cells via CRISPR/Cas9. The *Nipped-A* sgRNA target sequence consisted of the 20nt preceding and AGG PAM sequence situated five nucleotides upstream of the *Nipped-A* translation start. Two oligonucleotides for the sgRNA target were synthesized (Nipped-A Target F1-5′ ttcgAAGTTGTTGAACGAGTTAAA 3′ and Nipped-A Target R1-5′ aacTTTAACTCGTTCAACAACTTc 3′) that included overhangs (underlined) to allow ligation into pAc-sgRNA-Cas9 vector digested with *BspQI*^72^. Clones containing the sgRNA target insert were confirmed by sequencing. The resulting pAc_Nipped-A_sgRNA vector was used to generate stably transfected Nipped-AsgRNA S2 cells as described^68^. A donor vector containing N-terminal 3xHA-tagged *Nipped-A* was generated by amplifying genomic DNA via PCR to generate a 2009 bp left homologous arm (using primers LA_Forward 5′-CTT GCT GGG CCA CGA ACA GAA C-3′ and LA_Reverse 5′-**GAC GTC** TAA CTC GTT CAA CAA CTT GAC CG-3′) and a 2041 bp right homologous arm (using primers RA_Forward 5′-CGG TCA AGT TGT TGA ACG AGT TA**G ACG TC**-3′ and RA_Reverse 5′-TCC ATG GTC CTA CAT CTC TCG GTA CG-3′), which replaced the PAM sequence in the sgRNA target sequence with an *AatII* restriction site (bold). These arms were inserted into the TA plasmid (Life technologies) to form Nipped-A_donorAatII. 3xHA was inserted between the *Nipped-A* left and right homology arms by digesting with *AatII* and an *AgeI* restriction site present 20 bp downstream of the *Nipped-A* start codon and ligating annealed complimentary 3XHA-tag containing flanking oligos (5′- C ACA ATG TAC CCC TAC GAT GTG CCC GAT TAC GCC GGC TAT CCG TAT GAC GTG CCG GAC TAT GCC GGA GGC TAC CCC TAC GAT GTG CCC GAT TAC GCC TCG GTT ATT GAA AAT GTA -3′ forward and 5′- CCGGT AC ATT TTC AAT AAC CGA GGC GTA ATC GGG CAC ATC GTA GGG GTA GCC TCC GGC ATA GTC CGG CAC GTC ATA CGG ATA GCC GGC GTA ATC GGG CAC ATC GTA GGG GTA CAT TGT G ACGT -3′ reverse primers) into the *AatII* and *AgeI* sites to form the Nipped-Adonor3xHA plasmid. The Nipped-Adonor3xHA plasmid was transfected into Nipped-AsgRNA S2 cells, which were selected for recombination of the 3xHA insert into *Nipped-A* using 5 mg/ml of puromycin as described^72^. For transfection, 1 μg of gRNA vector and 1 μg of donor vector was transfected. HA-*Nipped-A* recombinants were identified by PCR using Nipped-A_HAinsert forward (5′ TGCATCTCTGTGGGAGCTCTA 3′) and reverse (5′ CGACGGATATGCCGGACTTT 3′) primers flanking the HA insert. Recombinants were then amplified by PCR using Nipped-A_5′flank forward (5′ AGGACAGCACGAGCACAAA 3′) and reverse (5′ AAGCCTTAGGCCGCATTACG 3′) primers near the 5′ end of the upstream flank and Nipped-A_3′flank forward (5′ TGGTGGACGGCAGTTTTCAT 3′) and reverse (5′ CGTGTAATCGTCGTCCTGCT 3′) primers near the 3′ end of the downstream flank to confirm that *Nipped-A* flanking regions were intact. Accurate insertion of the 3xHA tag at the N-terminus of *Nipped-A* was confirmed by sequencing.

### Generation of HA-tagged *Nipped-A* coding sequence in Drosophila

A 3xHA epitope tag was inserted at the N-terminus of the endogenous *Nipped-A* gene in Drosophila via CRISPR/Cas9. The Nipped-A Target F1 primer used to generate the pAc_Nipped-A_sgRNA plasmid above was used with the Nipped-A Target R2 primer (5′ aaacTTTAACTCGTTCAACAACTT 3′) that included an overhang (underlined) to allow ligation into the pCFD1: U6:1-gRNA vector digested with *Bbs1*^73^. The resulting pCFD1_Nipped-A_sgRNA plasmid was confirmed to contain the correct sgRNA target insert by sequencing. The Nipped-Adonor3xHA plasmid that was used to epitope tag *Nipped-A* in S2 cells was also used as the donor plasmid to add the 3xHA epitope tag to the endogenous *Nipped-A* gene in flies. The pCFD1_Nipped-A_sgRNA plasmid and the Nipped-Adonor3xHA donor plasmid were both injected to *vasa*-Cas9 (BDSC # 51324) flies to generate recombinant strains (BestGene). Injected *vasa*-Cas9 flies were crossed to *w*^1118^; + / + ; TM2/TM6B flies to generate F1 progeny, which were backcrossed to *w*^1118^; + / + ; TM2/TM6B flies to generate F2 offspring homozygous for the 3^rd^ chromosome from the injected flies. These offspring were screened for the presence of homologous 3X-HA tag recombinants at the N-terminus of *Nipped-A* by PCR using the Nipped-A_HAinsert primers as described above. The upstream and downstream flanking regions for all HA-*Nipped-A* recombinants by PCR using Nipped-A_5′flank and Nipped-A_3′flank primer sets as described above. HA-*Nipped-A* flies were sequenced to confirm the integrity of the 3X-HA tag and in-frame fusion to NIPPED-A.

### Circadian locomotor activity monitoring

Flies for each RNAi strain were crossed with *tim*-GAL4 and *pdf*-Gal4 to knock down expression in all clock cells or PDF-expressing LN_v_s, respectively. Progeny of these crosses (RNAi knockdown), and their respective controls (UAS-RNAi only, *pdf*-GAL4 and *tim*-GAL4 strains) were used to measure locomotor activity rhythms. Two to three day old male flies were entrained for three days in 12 h light:12 h dark (LD) cycles, and then kept in constant darkness (DD) for seven days at 25 °C. Locomotor activity was monitored using the *Drosophila* Activity Monitor (DAM) system (Trikinetics). DD activity data was analyzed using ClockLab (Actimetrics) software as described^74^. Statistical analysis of period length and strength levels were performed using one-way ANOVA, followed by Tukey multiple comparisons test with GraphPad Prism 5 (Prism, La Jolla, CA).

## Supplementary information

Supplementary Information 1

Supplementary Information 2

Supplementary Information 3

## Data Availability

The datasets and reagents generated during and/or analyzed during the current study are available from the Bloomington Drosophila Stock Center or the corresponding author upon reasonable request.
