# Supplementary material for: Proteomic analysis of Drosophila CLOCK complexes identifies rhythmic interactions with SAGA and Tip60 complex component NIPPED-A
Source: Sci Rep. 2020 Oct 21;10:17951. doi: 10.1038/s41598-020-75009-5 (PMC7578830; doi:10.1038/s41598-020-75009-5)
Supplement: Supplementary file 1 — Supplementary Information 1 [file 41598_2020_75009_MOESM1_ESM.pdf]

Supplementary Material

Proteomic analysis of Drosophila CLOCK complexes identifies rhythmic interactions with SAGA and Tip60 complex component NIPPED-A

Guruswamy Mahesh[1], Gustavo B. S. Rivas[1], Courtney Caster[1], Evan B. Ost[1], Ravi Amunugama[2], Richard Jones[2], David L. Allen[2] and Paul E. Hardin[1]*

[1]Department of Biology and Center for Biological Clocks Research, Texas A&M University, College Station, TX 77843; [2]MS Bioworks, LLC, Ann Arbor, MI 48108

*Corresponding author: Paul E. Hardin, phardin@bio.tamu.edu

Supplementary Figure S1. Relative levels of core clock proteins in GFP-CLK complexes.

Supplementary Figure S2. NIPPED-A interacts with CLK in S2 cells.

Supplementary Figure S3. RNAi knockdown of Nipped-A in clock cells reduces HA-NIPPED-A immunostaining in sLN_vs and LN_ds.

Supplementary Data S1. Proteins identified in GFP-CLK complexes at different times during a diurnal cycle.

Supplementary Data S2. Proteins identified in FLAG-CYC complexes at Zeitgeber Time 24

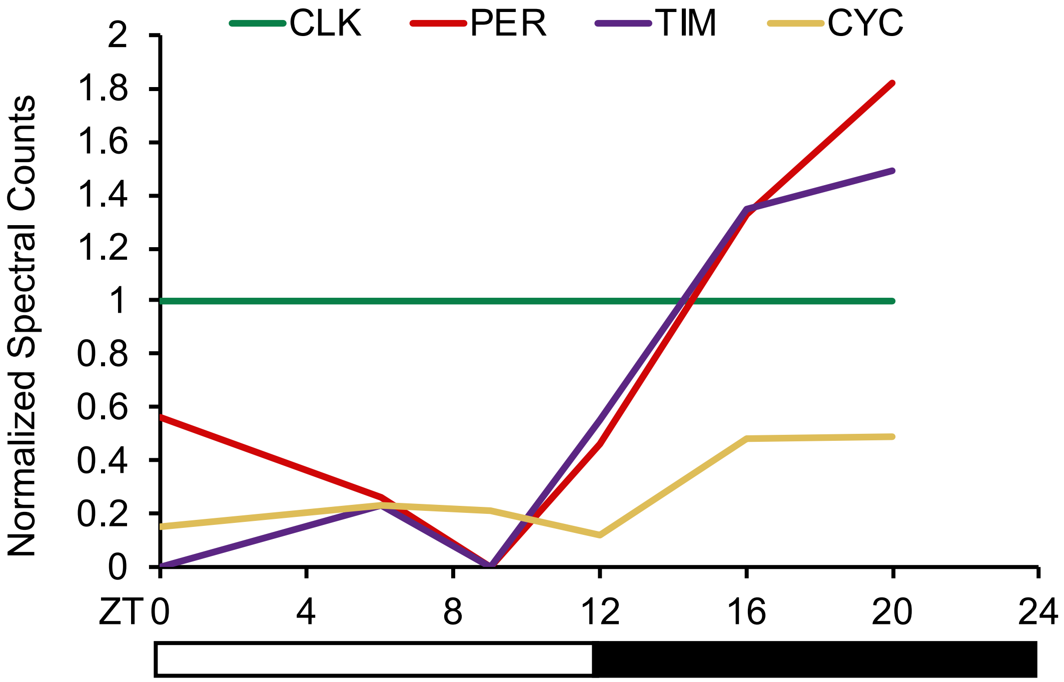

Figure S1. Relative levels of core clock proteins in GFP-CLK complexes. The number of spectral counts for CLK (green), CYC (yellow), PER (red) and TIM (purple) in figure 1A were normalized to CLK by dividing by the total number of spectral counts for CLK at each timepoint. The normalized spectral counts for each core clock protein are shown, where CLK level is 1.0. White bar, times when lights are on; black bar, times when lights are off.

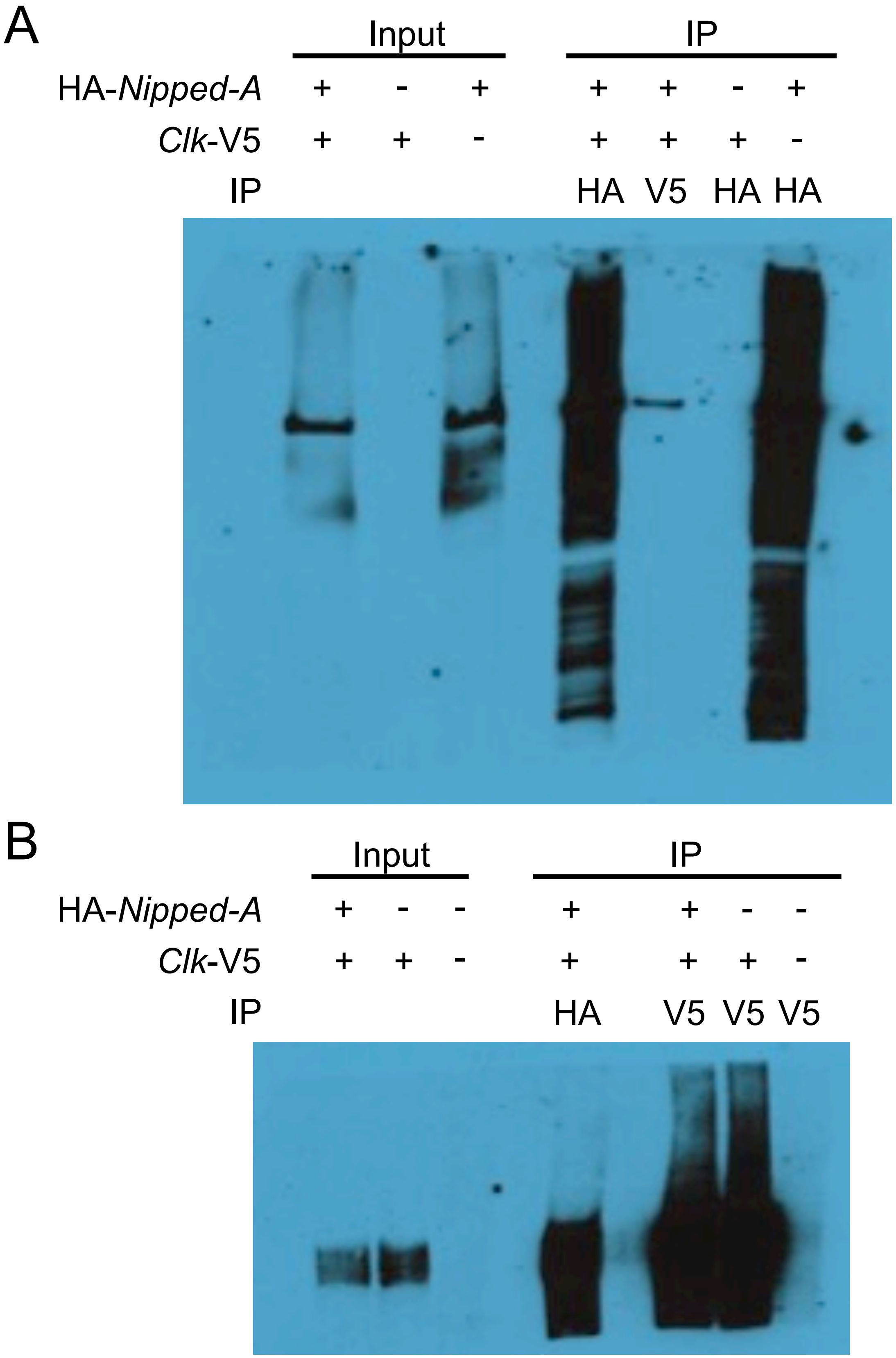

Figure S2. NIPPED-A interacts with CLK in S2 cells. (A) Full-length image of long-exposure western blot shown in Figure 3B. (B) Full-length image of long-exposure western blot shown in Figure 3C.

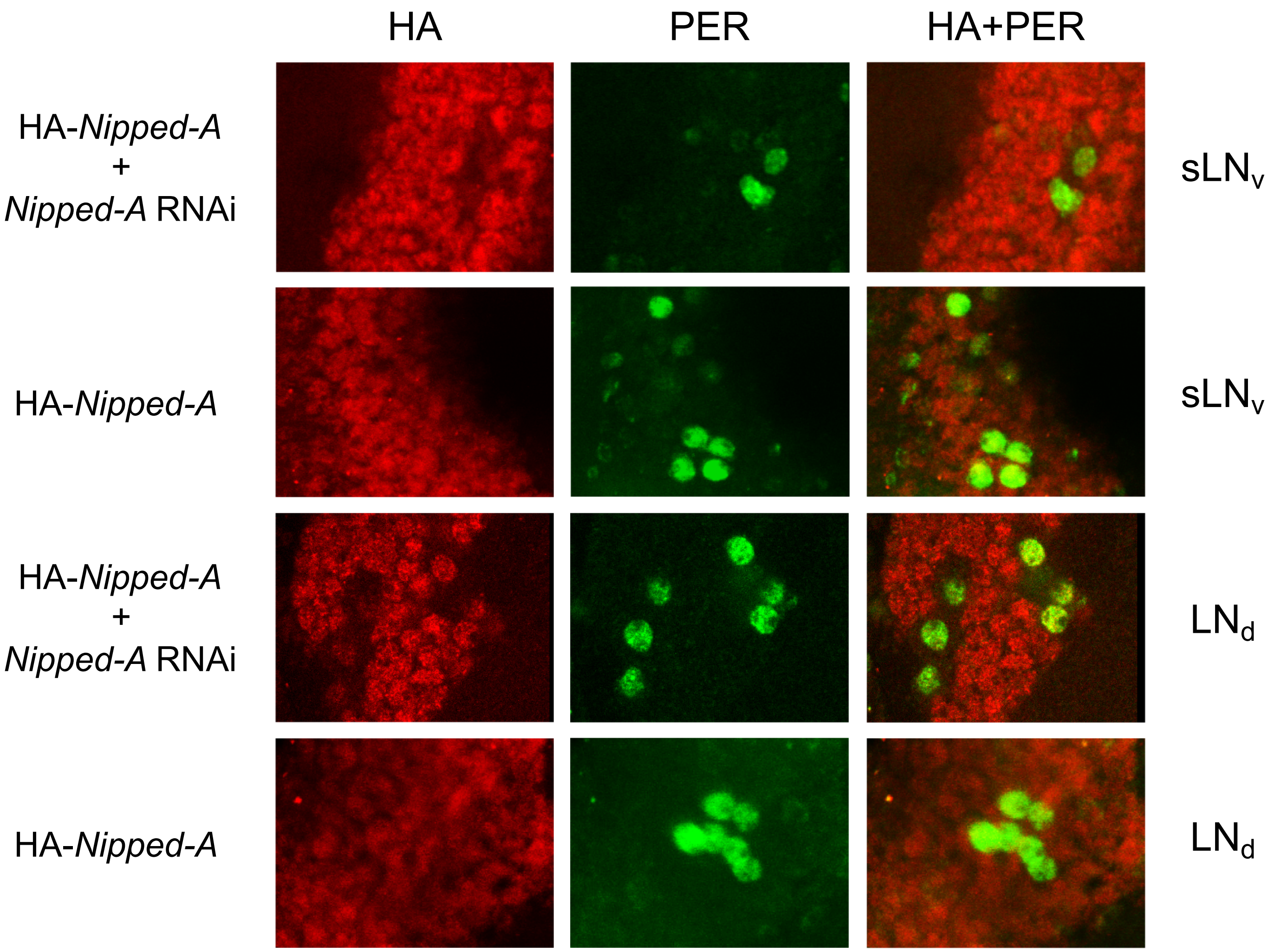

Figure S3. RNAi knockdown of Nipped-A in clock cells reduces HA-NIPPED-A immunostaining in sLN_vs and LN_ds. Brains from w^{1118}, UAS-*dicer*; 3x HA-*Nipped-A*/*tim*-Gal4; *Nipped-A* RNAi3/+ (HA-Nipped-A+Nipped-A RNAi) and w^{1118}, UAS-*dicer*; 3x HA-*Nipped-A*/+; *Nipped-A* RNAi3/+ (HA-Nipped-A) flies collected at CT24 were immunostained with HA and PER antibodies and imaged by confocal microscopy. PER (green), HA (red) and PER + HA (yellow) signals are shown for the sLN_v and LN_d groups of circadian pacemaker neurons. All images represent 24 or more brain hemispheres.
